# Strategies and approaches in plasmidome studies—uncovering plasmid diversity disregarding of linear elements?

**DOI:** 10.3389/fmicb.2015.00463

**Published:** 2015-05-26

**Authors:** Julián R. Dib, Martin Wagenknecht, María E. Farías, Friedhelm Meinhardt

**Affiliations:** ^1^Planta Piloto de Procesos Industriales Microbiológicos–Consejo Nacional de Investigaciones Científicas y Técnicas, Tucumán, Argentina; ^2^Institut für Molekulare Mikrobiologie und Biotechnologie, Westfälische Wilhelms-Universität Münster, Münster, Germany; ^3^Instituto de Microbiología, Facultad de Bioquímica, Química y Farmacia, Universidad Nacional de Tucumán, Tucumán, Argentina; ^4^Institut für Biologie und Biotechnologie der Pflanzen, Westfälische Wilhelms-Universität Münster, Münster, Germany

**Keywords:** plasmidome, circular plasmid, linear plasmid, extrachromosomal DNA, episome, metagenomics

## Abstract

The term plasmid was originally coined for circular, extrachromosomal genetic elements. Today, plasmids are widely recognized not only as important factors facilitating genome restructuring but also as vehicles for the dissemination of beneficial characters within bacterial communities. Plasmid diversity has been uncovered by means of culture-dependent or -independent approaches, such as endogenous or exogenous plasmid isolation as well as PCR-based detection or transposon-aided capture, respectively. High-throughput-sequencing made possible to cover total plasmid populations in a given environment, i.e., the plasmidome, and allowed to address the quality and significance of self-replicating genetic elements. Since such efforts were and still are rather restricted to circular molecules, here we put equal emphasis on the linear plasmids which—despite their frequent occurrence in a large number of bacteria—are largely neglected in prevalent plasmidome conceptions.

## Introduction

Ecological impacts of plasmids are beyond doubt. In a given environment such accessory genetic elements (when they have the capacity to integrate into the genome occasionally also termed episomes) commonly carry information that is—under given circumstances—beneficial for their prokaryotic host cells. A large number of plasmid-borne genes are known to permit survival, flexibility and adaptation (or durability) to environmental changes. Plasmid-encoded qualities include virulence factors, resistance to antibiotics, production of antimicrobials, degradation of xenobiotics, and functions involved in bacteria–host interactions (Smalla et al., 2000c). Moreover, those conferring conjugative capabilities facilitate horizontal gene transfer. Hence, plasmids are considered to play key roles in evolutionary events of a given microbial community (Koonin and Wolf, 2008).

Recording of extra-chromosomal genetic elements of bacterial populations from diverse environments includes culture-dependent or -independent approaches. While the former is self-explanatory, for the latter several methods have proven to facilitate detection and subsequent characterization of novel accessory genetic elements; such as the exogenous plasmid isolation by biparental matings (Bale et al., 1988). It relies on the transfer, replication as well as the expression of selectable markers, or triparental matings (Hill et al., 1992; Smalla et al., 2006) which is based on the ability of genetic elements to transfer small mobilisable plasmids carrying selectable markers into a new recipient. As anticipated, such methods selectively addressed and indeed disclosed conjugative and/or mobilisable plasmids (Smalla et al., 2000a). Moreover, PCR-based detection methods (Götz et al., 1996; Turner et al., 1996; Sobecky et al., 1998; Smalla et al., 2000b; Heuer et al., 2009; Jechalke et al., 2012) or in combination with Southern Blot hybridization (Smalla et al., 2006; Binh et al., 2008; Dealtry et al., 2014a,b), for specific sequences of mobile genetic elements are suitable for screening and abundance- or diversity estimations but may not allow to characterize plasmids as a whole or to elucidate the host(s) by their nature (Smalla and Sobecky, 2002; Heuer and Smalla, 2012).

**FIGURE 1:**
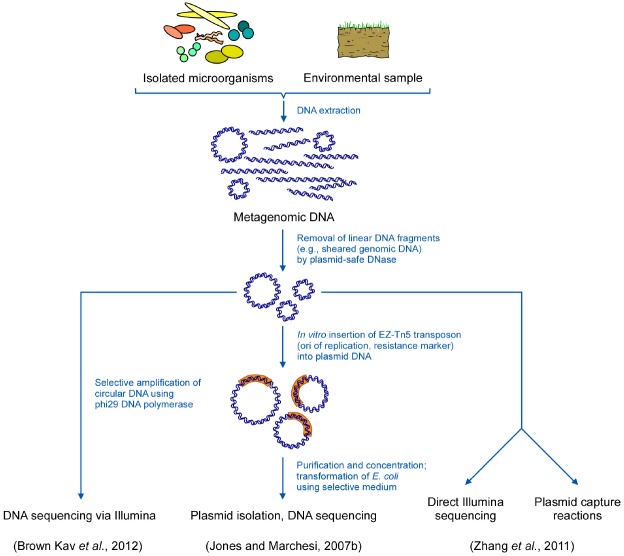
**Schematic representation of strategies for metagenomic studies of extrachromosomal DNA elements**.

The “Transposon-aided capture protocol” (TRACA, Jones and Marchesi, 2007a; see Figure 1) likewise proved to be a straightforward method for studying functions and, thus, ecological impacts of plasmids. Originally developed for recording plasmids from the human gut microbiota (Jones and Marchesi, 2007a), more recently, it has also proven successful in capturing accessory DNA from activated sludge (Zhang et al., 2011) as well as from human dental plaque (Warburton et al., 2011). TRACA allows for the acquisition of plasmids from a rather wide but—as a matter of fact—still limited range of bacterial species from environmental DNA preparations and their subsequent stable maintenance in surrogate host species.

Despite the undeniable potential of above culture-independent methods, there are intrinsic limitations and disadvantages, especially with respect to the incomplete number of elements that can be captured or isolated, necessarily leaving the unrecorded plasmid population unexplored (Jones and Marchesi, 2007b).

High-throughput sequencing technologies encouraged and accelerated not only the genetic studies of individual genomes but also allowed for the parallel investigation of the genetic material from diverse organisms in a given habitat to the sequence level. “Metagenomics,” i.e., sequencing and analysis of DNA isolated from environmental samples, is indeed not only a powerful tool for the genetic analysis of environmental issues but additionally proved useful for finding novel natural products and biotechnologically relevant new proteins/enzymes of non-cultivable or at least hitherto unknown organisms (Lorenz and Schleper, 2002; Daniel, 2004; Marchesi, 2012).

The analysis of the huge amount of data resulting from a metagenomic approach in general includes data pre-filtering, sequence assembly, gene predictions, determination of species diversity, and comparative alignments, and, thus, still poses a challenge. In fact, extraction of the useful biological information can occasionally cause confusion rather than clarity, as the analyzed environmental sample actually and almost necessarily consists of a mix of genetic material, originating not only from bacteria but also from other biological units such as yeasts, viruses, algae, protozoa, insects, worms, etc. (McHardy and Rigoutsos, 2007). Moreover, metagenomic samples not only include chromosomal DNA, but also all types of known and, by then, unknown accessory genetic elements. Due to the prominent role the latter have as evolutionary players and environmental agents, there are attempts to record separately the genetic information with respect to the total plasmid DNA sequences obtained in metagenomics, i.e., the plasmidome.

The plasmidome (a composite of plasmid and kingdom) refers to the entire plasmid DNA of an environmental sample independent of cultivation (the culture-independent plasmidome) but should—as a matter of course—include the elements isolated from bacteria that have been isolated and cultured (the culture-dependent plasmidome; Fondi et al., 2010; Bleicher et al., 2013; Brolund et al., 2013; Song et al., 2013). Consequently, in this manuscript we refer to the plasmidome as the entire plasmid community in a given environment that is most often resolved by metagenomic approaches during high-throughput-sequencing experiments.

Until the plasmidome concept appeared on the scene and became part of the “omics” family, metagenomic studies have clearly underestimated the role and impact of plasmids. This is mainly due to technical limitations or protocols that could have partially blinded out the plasmid origin of genetic information because commonly performed microbial community sequencing projects do not *a priori* separate chromosomes from plasmids. Indeed, a given metagenome is generally a mixture of chromosomal and plasmid sequences (Zhou et al., 2008), in which the relation of both vastly remains in favor of the chromosomes (Li et al., 2012).

Here, we deal with and review genomic studies addressing plasmid sequences obtained from environmental samples demonstrating the excessively frequent occurrence of such genetic elements in different habitats. Moreover, we emphasize linear plasmids which—despite their frequent occurrence in a number of bacteria—are still neglected in current plasmidome conceptions.

## Global Plasmid Studies in Cultured Strains

By applying the 454 sequencing technology the plasmid metagenome of cultivable antibiotic resistant bacteria from a wastewater treatment plant was determined (Szczepanowski et al., 2008). Bacteria from the sludge were exposed to selective conditions using ampicillin, cefotaxime, cefuroxime, ciprofloxacin, erythromycin, gentamicin, kanamycin, norfloxacin, rifampicin, spectinomycin, streptomycin, or tetracycline. From the viable, i.e., cultivable organisms, total DNA was harvested after alkaline lysis and, subsequently, plasmids were purified by caesium chloride density ultra-centrifugation to get rid of contaminating chromosomal DNA. Such total plasmid preparation was used as the template for sequencing. Assembling the reads yielded 605 contigs with a minimum length of 500 bases, which indeed predominantly referred to plasmid-borne sequences coding for survival functions and enzymes involved in transposition. As one may expect from the experimental set-up, several resistance-conferring sequences covering all major classes of antimicrobial drugs were also identified in such plasmid metagenomic data.

For recording the total plasmid content of a pathogenic *Salmonella enterica* strain isolated from pork meat (Bleicher et al., 2013), bacterial bulk DNA was prepared by applying a bacterial artificial chromosome (BAC) isolation protocol (Rondon et al., 1999) to reduce the risk of losing large plasmids. Indeed, the existence of four plasmids became evident which was confirmed by sequencing a clone library of DNA fragments that was generated from sheared DNA. Similarly, the global plasmid content of extended-spectrum beta-lactamase (ESBL) producing *Escherichia coli* was determined (Brolund et al., 2013). Several diverse ESBL-producing strains were selected and the purified plasmid DNA from each of the strains was subjected to high-throughput 454 sequencing, revealing at least 22 large plasmids as well as small cryptic high copy-number plasmids. Analysis of the sequencing data uncovered genes conferring resistance to the following groups of antibiotics: beta-lactam, fluoroquinolones, trimethoprim, sulphonamides, macrolides, aminoglycosides, tetracycline, and chloramphenicol. Overall, 19 resistance genes were identified in the plasmidome. Moreover, plasmids were also analyzed as to replicon type and total gene-content. Plasmids of the incompatibility group F were the most common, and only two were of incompatibility group I1.

The plasmidome of 106 strains of *Enterococcus faecalis* was characterized by determining the number of plasmid replicons (145), sizes (5–150 kb), replicon types (*rep*2, *rep*6, *rep*8, and *rep*9) as well as by antibiotic-resistance-conferring traits (erythromycin, tetracycline, and integrase gene; Song et al., 2013). As there is no sequencing data available such results need further confirmation as for the above outlined investigations.

A comparative analysis of plasmids and chromosome sequence data available for bacteria belonging to the clinically important genus *Acinetobacter* was recently performed by applying a novel bioinformatic tool, Blast2Network (Fondi et al., 2010). The study, as a pan-plasmidome approach, included all available completely sequenced *Acinetobacter* (circular) plasmids and chromosomes from the National Center for Biotechnology Information (NCBI). The authors not only suggested an evolutionary path for highly mobile genetic elements lacking extensively shared genes but also postulated that transposases as well as the selective pressure for mercury resistance apparently played a pivotal role in plasmid evolution in *Acinetobacter*.

Though the above mentioned plasmidome studies significantly contributed to the understanding of mobile genetic elements from a given bacterium or a bacterial group, the obtained data largely depend on the plasmid extraction procedures, and—linked to the former—the plasmid copy number. It necessarily excludes elements that are lost due to selective conditions. In addition—as a matter of fact—plasmids from non-cultivable organisms are out of reach.

## Culture-Independent Plasmidome

Prior to the plasmidome concept, that has its roots in the metagenomics (Kav et al., 2012), there were attempts to study the diversity of accessory elements in a defined environment (somewhat a kind of host-dependent plasmidome-independent from the culturability of the host), e.g., by the above mentioned “exogenous plasmid isolation” procedures. Since such approaches make use of biparental or triparental matings exogenous plasmid isolations have intrinsic limitations: Elements to be captured have to be conjugative (or mobilizable), must stably replicate, and conjugation is—at least to a certain degree-host specific (except for broad host range plasmids such as IncP-1 plasmids). However, though retrieval of information about the plasmidome of a specific environment obtained by applying such procedures is—inherent to the system—rather narrow, the technique was successfully applied to isolate plasmids conferring resistance to antibiotics or heavy metals (Hill et al., 1992; Lilley et al., 1996; Dahlberg et al., 1997; Drønen et al., 1999; Smalla et al., 2006; Binh et al., 2008; Heuer et al., 2009). Elements encoding degradative enzymes were obtained by transposon-aided capture (Jones and Marchesi, 2007a,b; Table 1) and—less abundant but efficiently transferring—broad host range plasmids were also isolated (Top et al., 1995).

**TABLE 1:** **Examples of exogenous plasmid isolation and transposon aided capture methods**.

**Method**	**Sample origin**	**Host(s) or receptor(s)**	**Size of plasmid(s) isolated (kb)**	**Plasmid-borne phenotypes**	**Reference**
EPI-BM	River epilithon	*Pseudomonas putida strep^r^*, Rif^r^, *Ilv-*, *Leu-*	165 MDa	Mercury and UV resistance	Bale et al. (1988)
EPI-BM	Marine bacteria	*P. putida* Rif^r^	∼60	Mercury resistance	Dahlberg et al. (1997)
EPI-BM	Rhizosphere of alfalfa	*Sinorhizobium meliloti*^1^	52–75	Mercury resistance	Schneiker et al. (2001)
EPI-BM	Soil	*Alcaligenes eutrophus Rif^r^*	63–97	Degradation of 2,4-D^2^	Top et al. (1995)
EPI-BM	Activated sludge	*Pseudomonas* sp. B13^1^	41–69	Mercury and Antibiotic resistances	Dröge et al. (2000)
EPI-TM	Epilithic microbial communities	*P. putida UWC5 (Rif^r^*, *Sm^r^*, *Trp-) recipient P. putida UWC3 (Rif^r^*, *Ilv-) donor*^3^	40–200	Antibiotic resistances, mercury resistance	Hill et al. (1992)
EPI-BM	Piggery manure	*E. coli* ^1^ Rif^r^	ND	Antibiotic resistances^4^	Binh et al. (2008)
EPI-BM	Soil	*E. coli* ^1^ Rif^r^	ND	Sulfonamide resistance	Heuer and Smalla (2012)
TRACA	Human dental plaque	*E. coli*	<8	Rep, integrase, mob, toxin/antitoxin system	Warburton et al. (2011)
TRACA	Activated sludge	*E. coli*	∼3	Putative beta-lactam resistance	Zhang et al. (2011)
TRACA	Human gut	*E. coli*	3–10	Toxin/antitoxin, phosphohydrolase/phosphoesterase	Jones et al. (2010)

EPI-BM, exogenous plasmid isolation by biparental matings; EPI-TM, exogenous plasmid isolation by triparental matings; ND, not determined. ^1^GFP-tagged. ^2^2,4-dichlorophenoxyacetic acid. ^3^Carring plasmid pD10. ^4^Amoxicillin, sulfadiazine and tetracycline.

Transposon-aided capture (TRACA, Jones and Marchesi, 2007a,b) is a culture-independent technique developed to address plasmid functions and their ecological impact. It was originally employed for the capture of plasmids residing in the human gut microbiota. TRACA allows the acquisition of plasmids from metagenomic DNA extractions from a wide range of bacterial species and their subsequent stable maintenance in a surrogate host species. Isolation of plasmids by TRACA is independent of functions encoded by the elements, such as selectable markers or the ability to mobilize and replicate in the surrogate host. By applying TRACA, plasmids (from both, Gram negative and Gram positive species) lacking conventional selectable markers, can be isolated and maintained in an *E. coli* host. A brief description of the procedure: Total DNA is extracted from the sample and the resulting bulk DNA is treated with a plasmid-safe DNase to remove sheared chromosomal DNA. The remaining mixture containing mainly intact circular molecules is then subject to an *in vitro* transposition reaction applying the EZ-Tn5 transposon that possesses an *E. coli* origin of replication and a selectable marker (*Ori*V/*Kan2*). Subsequently, the resulting circular hybrid elements are transformed into an *E. coli* surrogate host, followed by plasmid isolation and DNA sequencing (Figure 1).

Despite its unquestionable significance the method has some limitations such as gene inactivation by the transposon and the capture of mainly small plasmids (3–10 kb) which—in a given environment—may pretend that small plasmids are numerically dominant as they are preferentially captured (Warburton et al., 2011). Indeed, large plasmids can be hardly transformed into *E. coli* (Szostková and Horáková, 1998) and rather often they are present in low copy number. Furthermore, the host species cannot be identified and the efficiency of the method is influenced by the DNA quality as well as the integrity of the isolated plasmids. Plasmids which are unstable in *E. coli* or intractable by transposition as well as those present in low copy number can hardly be captured. Moreover, the capture of linear elements is totally excluded because the replication function included in the modified Tn5 does not have the ability to replicate linear plasmids, i.e., their termini. Replication of such DNA ends requires additional enzymatic functions (Warburton et al., 2011).

However, the potential of the technique became evident when “oral” metagenomic sequence data were compared with the isolated plasmid DNA and no homology was seen, proving that there is an unexplored genetic reservoir in the metagenome (Jones et al., 2010; Warburton et al., 2011).

With the TRACA system, 18 plasmids were captured from the human gut microbiota. They ranged in size from 3 to 10 kb with G+C contents (inferred mainly from the two totally sequenced pTRACA10 and pTRACA17) of 48.77–60.5% (Jones and Marchesi, 2007a). When four other elements (pTRACA18, pTRACA20, pTRACA22, and pTRACA30) from the same source with comparable sizes and G+C contents were also fully sequenced (Jones et al., 2010) it became evident that there are no homologous nucleotide sequences referring to these plasmids in available metagenomic data.

Also, when TRACA was applied for the isolation of bacterial extrachromosomal DNA from human oral plaque samples, obtained from patients suffering from periodontitis, 32 molecules were identified ranging in size from 0.9 to 7.3 kb, with G+C contents in the range of 30–52%. Again, these novel elements did not display any homology when compared to known metagenomic data (Warburton et al., 2011).

## Metagenomic Plasmidome

In metagenomic datasets from marine environments, “putative” plasmid sequences were identified along with their possible hosts (Ma et al., 2012). By applying bioinformatic tools plasmids were predicted to represent only 0.2–3% of all reads concomitantly displaying a high degree of variation. The majority of the plasmids were considered to be rather small and cryptic, encoding genes involved in replication, transfer, mobilization, stability, and partitioning. Nevertheless, due to the complex metagenomic information, the authors speculated that some contigs possibly are of phage origin or are presumably assembled as artifacts. Furthermore, some large contigs (comprising ∼300 kb) could not unambiguously assigned to a plasmid, and hence were suggested to be “accessory chromosomes” (Nierman et al., 2004). Besides, data might be biased toward identification of small plasmids, neglecting the larger ones as their assembly from metagenomic data would indeed require more sequencing data and/or larger reads.

A study to specifically explore the total plasmid community (including cultured and non-cultured bacteria) in a certain environment by next generation sequencing was first performed by Zhang et al. (2011). Total DNA was isolated from activated sludge by using a plasmid-specific DNA-purification kit to enrich extrachromosomal elements. Removal of remaining sheared genomic DNA from the samples was performed by applying an ATP-dependent plasmid-safe DNase. Subsequently, samples were either used for Illumina sequencing or plasmid capture by TRACA (Figure 1).

Sequencing reactions generated 11,550,210 clean reads comprising altogether 1.2 Gb. Annotations of the plasmid metagenome reads from the activated sludge sample revealed that the majority was of bacterial origin, dominated by Actinobacteria, Chloroflexi, Proteobacteria, Bacteroidetes, and Firmicutes; while little fractions came from fungi and protozoa. Mapping all the reads against the NCBI Plasmid Genome Database revealed matches with 307 different plasmids. Among such identified plasmids, pGMI1000MP and pA81 were identified as the most abundant elements. The latter plasmid was isolated from the haloaromatic acid degrading bacterium *Achromobacter xylosoxidans.* Its complete 98,192-bp sequence contained 103 open reading frames (ORFs) mostly encoding enzymes required for (halo)aromatic compound degradation or heavy metal resistance determinants (Jencova et al., 2008). pGMI1000MP is a megaplasmid (2,094,509 bp) often harbored in the soil-borne plant pathogen *Ralstonia solanacearum* (Salanoubat et al., 2002), which accounts for its high abundance in the sludge metagenome.

In addition, two plasmids were captured by TRACA (pST2 and pST10) and the comparison with all sequencing reads revealed that such elements were of high relative abundance and coverage. When the activated sludge resistome was determined as part of the plasmid metagenome, resistance genes for erythromycin and tetracycline as well as multidrug resistances were most abundant. In addition to plasmids, other mobile elements, such as integrons, transposons, and insertion sequences were predicted. Thus, the concerted application of TRACA and high-throughput sequencing resulted in reliable data revealing the situation of mobile genetic elements and the resistome in activated sludge of sewage treatment plants which is presumably close to reality.

When the bovine rumen plasmidome from 16 animals applying a metagenomics-based method was studied some modifications were included to improve both, quality and quantity of the total plasmid DNA to be sequenced (Brown Kav et al., 2012). Three different methods were applied to maximize lysis of diverse bacteria requiring unequal conditions (Brown Kav et al., 2013) and, in contrast to the previous method, plasmid isolations were performed directly from concentrated bacterial cell suspensions. Again, contaminating chromosomal DNA was removed using a plasmid-safe DNase that preferentially degrades linear DNA, and, additionally, circular DNA was amplified using Φ29 DNA polymerase to enrich plasmid DNA aiming at enhancing low copy number elements and to ensure quantities of plasmids allowing for sequencing. Finally, samples were subjected to deep sequencing via the Illumina paired-end protocol to enhance the *de novo* assembly process (Figure 1).

Roughly 34 million reads were generated and subjected to *de novo* assembly. Potential hosts of the plasmid-contigs were addressed as well. Most contigs were assigned to the domain Bacteria, with minor representations of the Archaea and Eukarya. The distribution of the dominant bacterial phyla within the rumen plasmidome was: Firmicutes (47%), Bacteroidetes (22%), Proteobacteria (20%), and Actinobacteria (9%). However, a significantly different phylum distribution in rumen microbiome was found. Furthermore, a functional analysis assignment of the rumen plasmidome was done and compared to those of the rumen, finding functions which are significantly enriched in the rumen plasmidome. Such functional comparison confirmed that the rumen plasmidome encodes more plasmid-specific functions and virulence factors than were detected in the rumen metagenome data sets (Walker, 2012).

An analogous approach (Li et al., 2012) addressed the plasmidome of a wastewater sample from a Danish sludge treatment plant. 200,000 sequencing reads with an average length of 300 bp were obtained. Analysis of the taxonomic distribution of BLAST hits showed plasmid sequences representing the phyla Actinobacteria, Proteobacteria, and Cyanobacteria. Additionally, plasmid-selfish traits as well as numerous novel putative plasmid replicases were identified. The apparent high abundance of small mobilisable plasmids was significant.

Considering the size range limitation of TRACA, the above strategy proved to be more efficient, revealing a tremendous increase of plasmid diversity and quantity concomitantly ensuring the coverage of large elements. In general, the metagenomic methods have several advantages when compared to the previous applied procedures used to study plasmids from communities such as the exogenous isolation or TRACA. In addition to the high throughput and cultivation independency, they reveal broad information on the extrachromosomal elements from the whole community, irrespective of encoded traits (but not from numerical dominances or sizes). Moreover, the metagenomic plasmidome may depict information about potential hosts, abundances, and resistance genes (resistome), and can easily be compared with classical metagenomic data from the same or similar environments. On the other hand, contamination with chromosomal DNA can impede the final data analysis and interpretation. As for any high throughput sequencing strategy, contigs assembly is challenging, and to obtain a complete sequence, especially for large elements or for plasmids with numerous repetitive sequences, is rather improbable. Moreover, the exonuclease digestion (by plasmid-safe DNase) and the whole genome amplification (by Φ29 DNA polymerase) applied for the preparation of plasmidome DNA favor small, un-nicked elements. It is, thus, not astonishing that mainly small plasmids (<10kb) are represented in high abundance in the plasmidome (Li et al., 2012). Nevertheless, a novel improved protocol, using an additional electroelution step, has enabled a more efficient capture of upper size range plasmids (>10 kb; Norman et al., 2014).

Plasmid peculiarities, such as high G+C contents, extensive secondary structures or repetitive sequences may affect sequencing reactions and/or reads assembly (Wagenknecht et al., 2010). Furthermore, modular composition of plasmids may obstruct the assembly of plasmid genomes from metagenomic reads, as identical or rather similar survival modules can be integral parts of different elements (Thomas, 2000; Szczepanowski et al., 2008).

Accessory plasmid segments are highly mosaic; they can be acquired from different sources and incorporated in the replicon by recombination. Hence, accessory plasmid regions are highly diverse and can potentially translocate into other molecules with the consequence that transposable elements and adjacent DNA segments can occur on different plasmids. Artificial assembly of contigs is, thus, not to be excluded.

## Linear Plasmids

Linear plasmids—as their circular counterparts—are extrachromosomal DNA elements. They have been found in a wide variety of both pro- and eukaryotic organisms. Firstly discovered in maize almost four decades ago (Pring et al., 1977), they do not only exist in higher plants, but also in filamentous fungi and yeasts, such as *Morchella conica* (Meinhardt and Esser, 1984) and *Kluyveromyces lactis* (Gunge et al., 1981), respectively. Among bacteria, linear elements are found in Gram-negative and -positive species (reviewed in Hinnebusch and Tilly, 1993; Meinhardt and Klassen, 2007). The majority, however, is found in the latter group, particularly in Actinomycetes including the genera *Streptomyces*, *Rhodococcus*, *Micrococcus*, and *Brevibacterium* (Dib et al., 2010a,b, 2013a,b,c). pSLA2 of *Streptomyces rochei* represents the first bacterial linear plasmid that was described (Hayakawa et al., 1979).

While eukaryotic linear plasmids are typically rather short, ranging in size from 1.1 kb (Düvell et al., 1988) to about 20 kb, such as pDP1 (18 kb) of *Debaryomyces polymorphus* (Fukuhara, 1995), bacterial elements are generally larger. They may reach lengths of several hundreds of kilobases, for instance pRHL2 (443 kb) of *Rhodococcus jostii* RHA1 (Shimizu et al., 2001). Extreme examples are pSCL4 (1.8 Mb) of *Streptomyces clavuligerus* ATCC 27064 (Medema et al., 2010) and the only 12-kb spanning pSCL1 of *Streptomyces clavuligerus* (Keen et al., 1988). Extremely large elements are frequently denoted as mega or giant linear plasmids.

Differences also concern the cellular localization. While in higher plants and filamentous fungi linear plasmids were exclusively found in mitochondria (reviewed in Meinhardt et al., 1990; Griffiths, 1995), in bacteria and yeasts a cytoplasmic localization is routinely realized. Among yeasts, pPH1 of *Pichia heedi* and pPK1 of *Pichia kluyveri* represent an exception, as they reside in the mitochondria (Blaisonneau et al., 1999). Moreover, the linear plasmid of *Chlamydomonas moewusii*, a green algae, is chloroplast-associated (Turmel et al., 1986).

**FIGURE 2:**
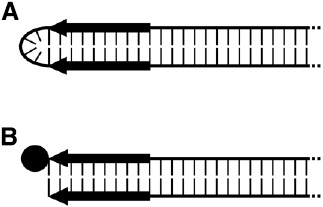
**Schematic representation of the termini of the two types of linear plasmids. (A)** Hairpin plasmid. **(B)** Linear plasmid with 5′-attached terminal protein (TP). Black arrows indicate the terminal inverted repeats (TIRs), and the TP is depicted as a filled circle.

Linearity of these elements unavoidably directs attention to the DNA ends, also referred to as telomeres. There are two types fundamentally different in structure. Based on such molecular differences, linear plasmids are grouped into hairpin elements and those with 5′-attached proteins (Figure 2).

Hairpin elements are characterized by terminal loops formed at each end of the plasmid due to covalent linkage of the two single DNA strands (Kikuchi et al., 1985). Moreover, each of the termini display short inverted repeats (terminal inverted repeats, TIRs). Elements of this type were found in representatives of the genus *Borrelia* (Hinnebusch et al., 1990; Hinnebusch and Barbour, 1991), such as *Borrelia hermsii* and *Borrelia burgdorferi*, which are known as causative agents of relapsing fever and Lyme borreliosis, respectively (Burgdorfer et al., 1982; Dworkin et al., 2002; Kobryn, 2007). Interestingly, the genomes of some bacteriophages of Gram-negative bacteria are organized as linear hairpin structures; among them are the *E. coli* phage N15 (Svarchevsky and Rybchin, 1984) and ΦKO2 of *Klebsiella oxytoca* (Stoppel et al., 1995). However, solely the corresponding prophages replicate as linear elements with hairpin ends (for details, see an excellent review by Hertwig, 2007).

The other type of linear DNA-elements possesses termini to which proteins (terminal proteins, TPs) are covalently attached to both of the 5′-ends. TIRs are likewise present. Members of this group of linear elements occur more frequently; they were found in a number of filamentous fungi, bacteria, and yeasts as well as in plants. Also, the genomes of some viruses and bacteriophages have TIRs and TPs (reviewed in Griffiths, 1995; Meinhardt and Klassen, 2007). Though phages with such genomic structure exist in Gram-negative bacteria, the 19.3-kb spanning TP-capped genome of phage Φ29 (Anderson et al., 1966; Bravo and Salas, 1997) of the Gram-positive *Bacillus subtilis* is the most prominent example.

Due to the identical genetic organization, the differentiation of such phage genomes and linear plasmids on a structural basis is almost impossible. Though the phage genomes are rather small compared to the majority of linear plasmids, size is not a suitable criterion, as small-sized linear plasmids likewise exist. The determination of an element’s nucleotide sequence along with the analysis of the encoded functions together with the genetic organization is necessary to differentiate plasmids from phage genomes.

The linearity of the TP-capped and the hairpin elements requires replication mechanisms which necessarily differ from those of circular plasmids. Indeed, elucidation of the replication process, the structures and the proteins involved, still pose a research challenge. Thoroughly investigated systems comprise elements from *Borrelia* and *Streptomyces* (Chater and Kinashi, 2007; Chen, 2007; Kobryn, 2007; Meinhardt and Klassen, 2007) but also rather recently *Arthrobacter* linear plasmids (Kolkenbrock et al., 2010; Wagenknecht, 2010; Wagenknecht and Meinhardt, 2011a,b).

As for their circular counterparts, linear plasmids may provide advantageous attributes to their hosts, many of them concern metabolic and physiological traits (Table 2) including catabolic gene clusters conferring the ability to degrade and metabolize a wide spectrum of organic compounds (Fetzner et al., 2007). Such catabolic linear elements are frequently found in soil bacteria, in particular in Rhodococci. Plasmid-borne resistances, allowing their hosts to tolerate heavy metals, such as arsenic and mercury, and antibiotics, have been reported, for a number of *Streptomyces* species, some Rhodococci, and in *Micrococcus* (Dib et al., 2010b). *Rhodococcus fascians* D188, a plant pathogen, harbors the linear plasmid pFiD188 coding for at least three key virulence determinants (Francis et al., 2007). The pathogenic capacity is a plasmid-linked trait in *Borrelia* too: Several pathogenicity determinants, instrumental in the infective cycle and required for antigenic variation, are plasmid-encoded (Girons et al., 1994). Some yeast linear plasmids code for a killer system (reviewed in Klassen and Meinhardt, 2007; Satwika et al., 2012). The toxin is secreted and kills or inhibits growth of competing yeasts. Few plasmids lacking a discernible phenotype, so-called cryptic or selfish elements, exist as well, particularly in filamentous fungi.

**TABLE 2:** **Compilation of selected actinobacterial linear plasmids and phenotypes attributed (modified after Wagenknecht, 2010)**.

**Plasmid**	**Host**	**Size (kb)**	**Phenotype attributed**	**Reference**
pAL1	*Arthrobacter nitroguajacolicus* Rü61a	113	Quinaldine metabolism	Overhage et al. (2005)
pAP13	*Brevibacterium* sp. Ap13	89	Repair of UV-induced DNA damage	Dib et al. (2010a, 2013b)
pNC30	*Gordonia rubripertincta* B-276 (formerly *Rhodococcus corallinus*)	∼185	Trichloroethene metabolism	Saeki et al. (1999)
pLMA1, pLMH5, pLMV7, pJD12	*Micrococcus* sp. A1, H5 and V7, D12	∼90–110	Antibiotic resistance^b^	Dib et al. (2010b, 2013a,c), Wagenknecht et al. (2010)
Unnamed	*Mycobacterium* sp. (six strains)^a^	∼110–330	Vinyl chloride metabolism	Coleman and Spain (2003)
pBD2	*R. erythropolis* BD2	210	Isopropylbenzene and trichloroethene metabolism, arsenite and mercury resistance	Dabrock et al. (1994), Kesseler et al. (1996)
pFiD188	*R. fascians* D188	∼200	Induction of fasciation	Crespi et al. (1992)
pHG201 pHG205	*R. opacus* MR11 and MR22 (formerly *Nocardia opaca*)	∼270 ∼280	Hydrogen autotrophy	Kalkus et al. (1990)
pHG204	*R. opacus* MR22 (formerly *N. opaca*)	∼180	Thallium resistance	Kalkus et al. (1993)
pRHL1 pRHL2	*R. jostii* RHA1	1123 443	(Polychlorinated) biphenyl and ethylbenzene metabolism	Masai et al. (1997); Shimizu et al. (2001)
SCP1	*Streptomyces coelicolor*	356	Methylenomycin synthesis	Bentley et al. (2004)
Unnamed	*S. fradiae*	420	Tylosin synthesis	Kinashi and Shimaji (1987)
pKSL	*S. lasaliensis*	520	Lasalocid A synthesis	Kinashi and Shimaji (1987)
Unnamed	*S. parvulus*	520	Actinomycin D synthesis	Kinashi and Shimaji (1987)
pSLA2-L	*S. rochei*	211	Lankacidin, lankamycin, and carotenoid synthesis	Hirochika et al. (1984); Suwa et al. (2000)
pRJ3L pRJ28	*Streptomyces* sp. CHR3 and CHR28	322 330	Mercury resistance	Ravel et al. (1998)
pSCL4	*S. clavuligerus* ATCC 27064	1796	Staurosporine, moenomycin, and beta-lactam antibiotic synthesis	Medema et al. (2010)
Unnamed	*S. venezuelae*	130	Chloramphenicol synthesis	Kinashi and Shimaji (1987)

*^a^These six* Mycobacterium *strains harbor linear plasmids, all of them conferring the ability to degrade vinyl chloride. ^b^The antibiotic resistance phenotype was demonstrated for pLMA1.*

As a distinctive feature, most of the linear plasmids originating from Actinobacteria are capable of conjugal transfer (Meinhardt et al., 1997; Chen, 2007). Hence, such bacteria may share genetic information and benefit from plasmid-borne attributes.

It is noteworthy to emphasize that the chromosomes of linear-plasmid-harboring bacteria may likewise be linear molecules. For example, the chromosomes of *Borrelia* species are—as for the corresponding linear plasmids characterized by hairpin telomeres (Casjens et al., 1997). Also, among Actinobacteria, in particular among the Streptomycetes which harbor TP-capped, linear extrachromosomal elements, a linear chromosome having covalently attached proteins at the 5′ ends is likewise realized. Moreover, more than a single linear plasmid may be present in the same host, as seen for the *Borrelia burgdorferi* type strain that harbors 12 different linear plasmids (Casjens et al., 2000; Sutton et al., 2000). A host cell may possess several coexisting linear and circular elements as well (Bentley et al., 2002; Casjens et al., 2000).

Linear elements are considered more flexible than circular ones; in particular the telomeres are considered to be prone for recombinational events (Volff and Altenbuchner, 2000; Chen et al., 2002). Intermolecular recombination may result into horizontal gene transfer, especially when the linearity of the chromosome along with the ability of conjugal plasmid transfer is taken into consideration as it not only may promote genetic exchange between plasmids but also between host chromosomes of compatible species.

## Linear Plasmids and the Plasmidome

Despite the commonness of linear plasmids in diverse microbial environments and despite their undeniable ecological importance (Meinhardt et al., 1990; Ravel et al., 2000; Ordoñez et al., 2009; Dib et al., 2010a,b, 2013a; see Table 2), these genetic elements were largely ignored in plasmidome studies. Indeed, metagenomic plasmidomes elide information originating from linear elements. While plasmid isolation strategies, such as the above endogenous or exogenous methods, in general can capture circular as well as linear elements, metagenomic plasmidome approaches disregard information carried on linear plasmids due to the applied experimental protocols: The isolation of extrachromosomal DNA in metagenomic plasmidome studies focuses on circular molecules and, moreover, the application of the (circular plasmid-safe) DNase decomposes, and thus eliminates, any kind of linear DNA as not only chromosomal fragments but also the linear plasmids are degraded.

Many linear genetic elements are rather large and may reach sizes many times higher than 100 kb, such as plasmid SCP1 (356 kb) from *Streptomyces coelicolor* A3(2) (Bentley et al., 2004). Hence, their isolation, purification and characterization require specific procedures (Dib et al., 2010a,b). In addition, TPs and TIRs conflict with the record of full length plasmid sequences (Parschat et al., 2007; Fan et al., 2012). Previous work on actinomycetal linear replicons showed that proteinase treatment of the TP-DNA sometimes leaves several residual amino acids bound to the DNA, preventing telomeric termini from being cloned (Hirochika et al., 1984; Huang et al., 1998; Goshi et al., 2002).

Moreover, other peculiarities of linear plasmids, such as excessive internal sequence repetitions or a high G+C bias, may require the combination of high-throughput sequencing and the conventional Sanger method to finally facilitate a reliable coverage and reads assembly (Wagenknecht et al., 2010).

Assuming that linear plasmids exist in a given environment, a plasmidome (as outlined above virtually restricted to circular elements) necessarily depicts only a partial representation of the extrachromosomal genetic elements. Taken into consideration the rather often large size of the non-recorded linear element(s), such as pSCL4 (1.8 Mb, Medema et al., 2010), the narrowness of the available genetic information in current plasmidome studies becomes evident.

Thus, even though a panoply of linear plasmids from isolates originating from diverse environments have been fully sequenced and genetically characterized (Le Dantec et al., 2001; Stecker et al., 2003; Bentley et al., 2004; Parschat et al., 2007; Dib et al., 2013b,c; Miller et al., 2013), the ecological impact of such accessory genetic elements as a whole remains largely obscure.

Up to the present there is no metagenomic plasmidome available that covers comprehensive information on linear genetic elements, which is, as outlined above, mainly due to the lack of an adequate experimental protocol. First and foremost, when it comes to linear elements, the application of a DNase while preparing the DNA for sequencing is of course prohibited, and—as linear plasmids can be very large—extraction protocols should additionally be adapted to cover a wide range of (big) sizes. Moreover, separating linear from circular plasmids constitutes another rather difficult task. Different running conditions during pulse field gel electrophoresis may help to distinguish linear from circular elements in samples containing a mix of linear and circular molecules as for a *Brevibacterium* strain that was found to harbor two differently structured large elements, i.e., pAp13 (linear) and pAp13c (circular; Dib et al., 2010a). However, in a complex mix of molecules, such as a metagenomic-plasmid type DNA sample, separation of molecules according to structural differences needs experimental skill and experience.

The determination of the abundance and diversity of circular plasmids in environmental samples was performed by applying PCR-based techniques coupled with *Southern* blots or quantitative reactions (qPCR). Since the methods make use of conserved sequences such approaches presumably can likewise be satisfactorily applied for studying the impact of linear plasmids on the ecology in a given environment. However, the number of sequenced linear plasmids is by far not comparable to that of the circular ones, and hence, defining backbone sequences for types or groups of linear elements is still in its infancy. Since, we found rather conserved plasmid specific functional regions in diverse linear elements from *Micrococci* isolated from different habitats (own unpublished results) there is hope for a future successful inclusion of linear plasmids in metagenomic plasmidome approaches by making use of the information originating from the shared modules.

On account of the hitherto known distinctive and unique characteristics of linear extrachromosomal genetic elements, studying the “linear” plasmidome is presumably well suited to provide deep insights into the ecological impact of such elements and will certainly add significant knowledge to the plasmidome in general.

### Conflict of Interest Statement

The authors declare that the research was conducted in the absence of any commercial or financial relationships that could be construed as a potential conflict of interest.
